# Death of Medical Editors

**Published:** 1854-03

**Authors:** 


### DEATH OF MEDICAL EDITORS.

---

We record with pain the death of Dr. Hester, Editor of the "New Orleans Medical and Surgical Journal." Dr. H. died of epidemic cholera on the 1st of December last, aged forty years. In 1844, in conjunction with Dr. E. D. Fenner, he established the journal mentioned, and after the withdrawal of Dr. F., he continued to conduct it alone, with eminent ability and success, up to the time of his lamented death. He was a man of high character, an able, independent journalist, an acute, forcible writer, and a physician greatly esteemed. The New Orleans Journal, for which he labored with uncommon industry, has acquired a wide popularity, and been productive of the happiest results to the profession.

Dr. Richard L. Howard, Professor of Surgery in Starling Medical College, and Editor of the "Ohio Medical and Surgical Journal," died of pneumonia, consequent upon Bright's disease, on the 15th of January, in the forty-fifth year of his age. His health had been declining for many months, and about a year ago albumen was detected in his urine. Dr. H. was a popular and instructive teacher, and as an Editor was courteous, dignified, and judicious. He did not write a great deal, but what he did produce gave evidence of a strong, discriminating mind. He was much beloved by his pupils, and maintained among men the character of a pure, upright man. Struggling in early life with poverty and many adverse circumstances, he rose to a position where ease, honor, and wealth seemed within his reach, but at a moment when life was most full of promise untimely death came to blight all his earthly prospects.

In the death of Dr. HESTER and Dr. HOWARD, our profession has lost two valuable and honored members, and the editorial corps two of its ornaments. Dr. HESTER is succeeded in the editorship of the N. O. Medical Journal by that learned and original writer, Dr. BENNET DOWLER, and Dr. JOHN DAWSON. also a writer of great industry and learning, takes charge of the Ohio Medical Journal. We hardly know the equals of these gentlemen for patient, induring application, and with such attainments as they have made in their profession, and such practice as they have acquired as writers, added to their laborious habits, there is reason to expect that these journals will attain a still higher standing under their direction.

